# Inequities Uncovered: Examining Exclusion in Indian Health Insurance Policies

**DOI:** 10.3390/healthcare14131885

**Published:** 2026-06-28

**Authors:** Dipika Jain

**Affiliations:** Jindal Global Law School, O. P. Jindal Global University, Sonipat 131001, India; djain@jgu.edu.in

**Keywords:** India’s healthcare system, timeline of public health in India, health insurance, health insurance policies in India, health insurance exclusions, age-based exclusions, gender-based exclusions, pre-existing ailment exclusions, constitutional violation

## Abstract

**Objective**: This study identifies specific patterns of exclusion in health insurance policies in India, based on age, gender and pre-existing ailments. **Method**: For its purposes, extensive primary research was conducted across different levels of the health insurance infrastructure, spanning the national health insurance scheme, 14 state health insurance policies, and individual and group health insurance policies offered by four government and five fourteen state health insurance policies as well as individual and group health insurance policies offered by four government and five private companies. In addition, a secondary literature review was conducted on topics such as health insurance, health financing, universal access to healthcare, and legal and regulatory frameworks. **Results**: The findings reveal that health insurance policies fail to address the distinct health needs of specific groups, such as women, children, and older people, and the concerns about their access to healthcare. Age-based exclusions demonstrate that the mere existence of specific policies for “seniors” is not sufficient to achieve the goal of universal health coverage in India. Health insurance policies for women lack coverage for reproductive and other health services, including gender affirming procedures. These exclusions can signify that such health services are unimportant and can be ignored in the insurance policies being offered by both the government and private companies, exacerbating the discrimination faced by groups such as persons with the capacity for pregnancy, as well as transgender persons. Finally, most health insurance policies covered in this study exclude congenital external anomalies as “pre-existing ailments”, while others impose specific waiting periods before coverage begins. **Conclusions**: This study identifies specific patterns of exclusion in both private and public health insurance policies in India, including those based on age, gender, and pre-existing conditions, to assess how these exclusions affect access to healthcare for specific groups.

## 1. Introduction

Universal Health Coverage (UHC) [1] ensures access to a full range of health services without financial hardship due to high out-of-pocket expenditures [2]. Access to essential healthcare services (the cornerstone of UHC) consists of the following components: Acceptability, or the willingness of people to avail of health services; Financial Affordability, which depends on people’s purchasing power and jurisdictional status of health financing; and Physical Accessibility, or the ability of persons to reach healthcare facilities providing the services they need [2]. Insurance is therefore a crucial component in ensuring access to healthcare, reinforcing citizenship rights, and promoting overall economic and social security.

In India, insurance was nationalised in 1956, six years after independence from the British Colonial government via the Life Insurance Corporation Act, 1956, which established the Life Insurance Corporation (LIC), a government corporation exclusively dealing in life insurance matters. This was followed by the General Insurance Act, 1972, which laid the foundation for the General Insurance Company (GIC) [3]. However, insurance privatisation began in the late 1990s with the advent of liberalisation. The Malhotra Commission was established in 1994 to recommend reforms in the insurance sector and advocated for private insurance providers [4]. As a result, the Insurance Regulatory and Development Authority (IRDA) Bill, 1996, was proposed [5]. The Bill ended the nationalisation of insurance and amended the Life Insurance Act. It allowed private companies to provide insurance and established the IRDAI [3]. The IRDAI was established to protect insurers and regulate insurance providers [3]. The IRDAI also allows private insurance providers to enter the market to improve access to insurance policies. The 2019 IRDAI Guidelines are binding on insurers and uniformise exclusions across private and public insurance policies. However, alarmingly, both private and public insurance policies include specific exclusions that can significantly affect access to timely and effective healthcare.

Recent scholarship has looked at exclusions based on gender, age, and chronic illness. Although public insurance schemes seem inclusive, indirect exclusion of certain groups manifests through specific design and implementation practices [6,7]. Socio-economic barriers arising from social and familial inequalities, lack of reproductive healthcare services, and gendered social mores that affect women’s mobility and financial autonomy have a disproportionate impact on their ability to access timely and affordable healthcare services [7].

Older adults remain underserved under public insurance schemes, which prioritise hospitalisation over geriatric, outpatient, or long-term care services [6]. There is a dearth of literature on age-based exclusions in insurance policies, despite evidence that older populations utilise health insurance far less, even though their needs are greater.

Exclusions based on pre-existing and chronic conditions are highly prevalent across both public and private insurance systems. Firstly, schemes do not adequately cover outpatient care and chronic disease management, exposing households to substantial financial risk [8]. Further, coverage of mental health conditions is limited, with mental illness, substance abuse, or suicide-related treatment being excluded or provided subject to lengthy waiting periods [9].

Despite substantial scholarship on PMJAY and related schemes, important gaps remain in the literature. First, most studies focus on patterns of insurance utilisation and outcomes, rather than examining the contents and frameworks of insurance contracts. Further, research on policy clause analysis, waiting periods, exclusion criteria, and age-based conditions that shape access to healthcare is sparse. Gender-based exclusions are documented in studies, but only in descriptive terms. Studies have identified that women utilise less insurance but do not delve into the role of policy design, through exclusions of reproductive healthcare services and tertiary-care orientation [7]. Research on age-based exclusions shows a similar pattern, with studies showing lower insurance utilisation among older adults, but no inquiry on age-based premiums, enrolment restrictions, and structural barriers to geriatric care [6]. There is also no comparative analysis of how insurance schemes treat chronic and pre-existing conditions. While scholars recognise the inadequacy of chronic care coverage, there is a dearth of comprehensive research on excluded conditions across public and private schemes [8].

Therefore, most literature on health insurance in India examines gender, age, or chronic illness separately, rather than analysing how different exclusions compound healthcare access issues [6,7]. There is no focused analysis of exclusions, which may affect vulnerable populations. For instance, older women managing chronic illness may simultaneously face gendered barriers, age-based exclusion, and condition-specific limitations within insurance systems.

India’s increased focus on insurance-driven healthcare financing has elevated the prominence of health insurance within the broader UHC framework. Aside from improving insurance enrolment, limited attention has been paid to insurance policy exclusions that reflect and perpetuate societal hierarchies. Insurance policy exclusions that relate to age, gender, sexual and reproductive healthcare, outpatient services, disabilities and pre-existing ailments significantly impact access to meaningful healthcare services by insured people. Given that these exclusions are applied uniformly across the board, marginalised insured persons are still likely to face inequitable access to healthcare.

India’s healthcare system is presently characterised by a large but weak public healthcare system working alongside an unregulated, profit-driven private healthcare system [10]. Tax revenues finance the public healthcare system and allocate its budget through inter-state budgetary allocation. The private healthcare system works on an out-of-pocket payment model for households [11]. In addition to these two health models, healthcare is also provided through the Central Government Health Scheme (CGHS) for government employees at the national level, and the Employee State Insurance Scheme (ESIC) for employees of establishments where more than 10 persons are employed, who draw a fixed salary of less than INR 21,000 a month, under the Employees’ State Insurance Act, 1948 [12]. Overall, public expenditure on health care in India is very low, at about 1.8% of the country’s GDP [13].

Within the public healthcare system, India has a government health-care infrastructure composed of 3 main pillars—subcentres (1 for every 5000 persons), primary health care centres (1 for every 30,000 persons), and community health centres (1 for every 120,000 persons) [12]. There is significant variation across states in the quality of these centres’ infrastructure and the health care they provide [14].

Despite this public healthcare infrastructure, about 70% of healthcare and healthcare spending in India are provided by the private sector [15]. Survey data from a 2014 study by the National Sample Survey Organisation (NSSO) showed that about 70% of hospitalisations in urban areas and 60% in rural areas occur in private-sector hospitals, leading to substantial out-of-pocket expenditure [15]. Out-of-pocket expenditure (OOPE), a major source of health financing in the country, contributes to 43.89% of total health expenditure [16]. An estimated 50 to 60 million people are pushed into poverty each year because of medical-related expenditure [17].

This article aims to identify the specific patterns of exclusion of persons based on age, gender, and pre-existing ailments across health insurance policies in India. Age-based exclusions include certain age-related diseases or conditions that are more likely to affect those nearing a specific age, such as arthritis and cataracts. Gender-based exclusions include exclusions related to maternity, pregnancy, sterility, fertility/infertility, assisted reproductive services and contraception. Pre-existing conditions impose specific waiting periods before coverage takes effect. Further, the analysis demonstrates that many health insurance policies exclude vaccinations and immunisations, as well as growth hormone therapy, which particularly affects infants and young children and can hamper their growth and have a serious, long-lasting impact on their health. Some policies also excluded congenital external anomalies, and others imposed a minimum 48-month waiting period from the policy start date. Categories of age, gender and pre-existing ailments often intersect across policies as well. Finally, the article analyses the disproportionate impact of exclusion on persons belonging to demographics, such as those of a particular age or belonging to a particular age bracket (infants and young children or the elderly), women (including persons with the capacity for pregnancy) and persons suffering from pre-existing ailments, while also examining their access to healthcare within a Constitutional framework of right to health.

## 2. Background

### 2.1. Healthcare Policy Reforms in India

To comprehend insurance policies and their exclusion frameworks, it is imperative to briefly understand the history of insurance-based reforms in India to establish a contextual foundation. Tracing the history of healthcare reform in India leads us to two big interventions. Firstly, in 2005, the National Rural Health Mission (NRHM) was launched to strengthen public health infrastructure in rural areas [18]. Health infrastructure, such as buildings, equipment, access to medicines and vaccines and human resources, was built or strengthened. NRHM emphasised primary health care, including maternal and child health, immunisation, and nutrition, leading to substantial gains in infant and maternal mortality indicators [19].

Secondly, in 2008, the Rashtriya Swasthya Bhima Yojana (RSBY), a central government-financed health insurance scheme targeting people living below the poverty line, was launched. Under this program, eligible groups would have access to cashless treatment in public and partnered private hospitals, up to INR 30,000 [20,21]. While the program increased healthcare access across various parts of the country, it ultimately failed due to operational inefficiencies, limited coverage of the target population, and significant inequities in coverage [22].

Finally, in 2017, the new draft of the National Health Policy brought three major shifts. These included the strategic purchase of healthcare services from the private sector to achieve UHC, the shift from narrow, selective primary health care to comprehensive primary health care provision in health and wellness centres, and the provision of free diagnostic and emergency care [23,24]. In this context, the Indian Government announced the Ayushman Bharat National Health Protection Mission (AB-NHPM), a centrally sponsored health protection programme, in 2018. The initiative offers hospital coverage to 40% of the country’s poor or low-income population. It aims to bolster preventive and promotive health care by revamping existing health care centres into health and wellness centres. The Government also established the National Health Authority to oversee the implementation of the AB-NHPS [25]. The second major initiative was the launch of the Intensified Mission Indradhanush 2.0 to achieve 90% vaccination coverage for children under two years of age [21]. The final notable reform was the establishment of the Health Technology Assessment in India to evaluate existing and emerging medical technologies, which would operate under the Department of Health Research.

#### Ayushman Bharat—PMJAY

Ayushman Bharat was recommended by the National Health Policy, 2017, and launched to achieve the vision of universal health coverage in India and meet the Sustainable Development Goals. The Pradhan Mantri Jan Arogya Yojana (AB-PMJAY), a nationwide publicly funded insurance scheme, was launched in 2018 [26].

AB-PMJAY is the world’s largest cashless health assurance scheme, providing a health cover of up to INR 500,000/- per family every year and access to 1670 health benefit packages across a network of over 25,000 empanelled hospitals and healthcare providers in the country [26]. It focuses on poor and vulnerable people who typically lack access to health insurance. The identification of eligible households under AB-PMJAY is based on the latest Socio-Economic Caste Census data for both rural and urban areas [27]. The National Health Authority (NHA) is the apex body responsible for implementing AB-PMJAY and is an attached office of the Ministry of Health and Family Welfare [25].

Although it appears that the AB-PMJAY does not impose restrictions based on “family size, age or gender” and that it includes “all pre-existing conditions” from the first day of coverage [26], it does involve certain exclusions (discussed below). To implement AB-PMJAY across states, State Health Agencies (SHAs), in the form of trusts or societies, have been set up by various states in India. SHAs have full operational autonomy in implementing the scheme in a particular state [25].

Beyond the Introduction and Background, Part 3 outlines the methodology. Parts 4 and 5 present the data analysis, identify patterns of exclusion across policies, and examine their implications for marginalised persons, particularly regarding the constitutional right to health and access to insurance, highlighting the gap in the realisation of UHC. Part 6 concludes the study by summarising exclusion patterns, discussing the implications on access to healthcare services and providing policy recommendations.

### 2.2. Literature on Health Insurance and Universal Health Coverage in India

Universal Health Coverage (UHC) refers to ensuring that people can access healthcare services without financial hardship [28]. In India, there are high levels of out-of-pocket expenditure (OOPE), limited reach of public health infrastructure, and a prominent, unregulated private healthcare industry [11]. Only 1.8% of India’s GDP is spent on healthcare, resulting in high dependence on private providers for service delivery and widespread infrastructure [6,8]. Therefore, health-related expenses still drive millions of Indian households into poverty on an annual basis [7,29].

India has therefore increasingly emphasised insurance as part of healthcare, adopting various publicly funded health insurance schemes (PFHISs), including the Rashtriya Swasthya Bima Yojana (RSBY), introduced in 2008, and the Ayushman Bharat Pradhan Mantri Jan Arogya Yojana (AB-PMJAY), introduced in 2018 [29,30]. These schemes are designed to reduce OOPE and increase access to healthcare for marginalised groups. However, despite increased insurance coverage across the population, scholars maintain that inequitable access, use and financial protections still prevail [31,32].

#### 2.2.1. Historical Evolution and Politics Around Public Health Insurance

The literature on the emergence of health insurance shows that political and electoral agendas primarily catalysed it. RSBY was introduced primarily as a social security initiative for unorganised sector workers, following formal recommendations from the National Commission for Enterprises in the Unorganised Sector [33]. The administration of the scheme by the Labour Ministry rather than the Health Ministry before the 2009 elections could have reflected systemic distrust of the public healthcare sector, resulting in priority given to insurance-based healthcare models involving private providers [29]. Further, at the time of PMJAY’s emergence, before the 2019 general elections [30,32], insurance-based healthcare had already become a systemic part of Indian health policy discourse [30]. Scholars also note that PMJAY’s market-oriented and ‘technocratic’ framework was influenced by NITI Aayog, the Prime Minister’s Office, as well as the WHO and World Bank [30]. The National Health Authority (NHA) was established as a quasi-autonomous body to implement PMJAY quickly. Still, concerns about accountability and legal legitimacy were raised because the scheme was not enacted by Parliament [30].

#### 2.2.2. Characteristics and Structural Considerations

PMJAY is recognised as one of the largest publicly funded health insurance schemes in the world, providing annual hospital coverage of up to INR 500,000 (Indian Rupees Five Hundred Thousand only) for vulnerable households identified through the Socio-Economic and Caste Census [31]. Notably, PMJAY does not impose any restrictions on insurance coverage based on age, gender, or family size [31], but the scheme’s design has come under scrutiny from scholars.

Firstly, the literature shows the conflict between insurance-based healthcare financing and public healthcare improvement. Scholars have argued that Thailand and China’s UHC frameworks, along with other successful systems, have elements of both insurance expansion and investment in public healthcare and primary care programmes [8]. However, in India, the citizens still relies heavily on private service delivery due to a lack of investment in public healthcare [31,32], and PMJAY, along with its associated public–private partnership models, is said to have hastened privatisation by directing public funding towards corporate hospitals [32].

The Ayushman Bharat programme established Health and Wellness Centres (HWCs) to improve primary healthcare. Still, the funds allocated were much lower than those for PMJAY, indicating an institutional preference for insurance-based private healthcare rather than primary healthcare and preventive services [32]. The UHC strategy in India, therefore, shows a clear prioritisation of tertiary healthcare rather than primary healthcare financing and improvement [8].

Secondly, PMJAY’s expenditure has become a cause for concern, with some scholars stating that expenses may exceed budgetary allocations, as expansion takes place [31,32]. Unless India increases public health investment, at least to 2.5% of its GDP as targeted by the National Health Policy, PMJAY is likely to face challenges in financial sustainability [32].

#### 2.2.3. Equity in Access and Utilisation of Healthcare Services

Inequity in healthcare access and utilisation across India is well-documented by researchers, with caste, class, gender and geography playing critical roles, despite PFHISs expanding formal insurance coverage. States like Tamil Nadu and Kerala, which have more robust healthcare infrastructure, show higher utilisation rates, while Bihar and Uttar Pradesh (with less robust infrastructure) show much lower utilisation. Unequal access and utilisation stem from factors such as the uneven distribution of covered hospitals, poor administrative services and low awareness levels amongst poorer groups [33].

Inequity in access and utilisation of health insurance is also highly gendered, with women showing much lower utilisation, despite being eligible under schemes and even formally enrolling. For instance, women constituted only 36% of beneficiaries under the CHCHIS scheme in Tamil Nadu and received much lower claim amounts than men [7]. Caste and class-based exclusion also persist, with Dalit and Adivasi groups facing discrimination in healthcare institutions and a lack of access to formal care, leading to reliance on informal health service providers [8]. Marginalised castes and poor households incur high OOPE because their insurance schemes end up disbursing very low claim values that do not cover treatment costs [8].

As stated earlier [33], poor awareness of insurance programmes’ benefits significantly affects utilisation, with a study in Gujarat showing that despite progressive awareness of PMJAY’s existence, poor awareness of its benefits reduced utilisation rates [34]. Similarly, despite widespread awareness of health insurance in Bihar, utilisation remained low due to complex administrative procedures, documentation, and poor awareness-building [35].

#### 2.2.4. Financial Protections and Out-of-Pocket Expenditure (OOPE)

A cardinal principle of UHC is ensuring people have access to healthcare services without financial hardship. Accordingly, a core objective of publicly funded health insurance is to reduce OOPE. However, the literature shows that insured households still incur significant OOPE due to insufficient reimbursement, leading to consistent rates of distress financing [8]. One reason is the exclusion of outpatient services and chronic disease prevention and treatment from PMJAY, which form a significant part of OOPE [33]. This results in affected households facing financial hardship and having to make OOP payments in hospitals, despite being eligible for insurance [31]. No country has achieved UHC by relying on OOP payments alone [28], as public investment, state buy-in, and consistent regulation of healthcare systems are necessary to achieve this goal. In India, limited government expenditure and reliance on the private sector result in continued financial vulnerability of populations [8].

#### 2.2.5. Ethical Concerns and Regulatory Issues

The literature explores ethical risks in the implementation of publicly funded health insurance schemes, indicating that supplier-driven demand, unnecessary add-on medical procedures, and poor accountability structures are common issues [6]. A study on RSBY implementation showed that in some hospitals, unnecessary surgeries were sanctioned and conducted to increase insurance claims [6]. Furthermore, private hospitals can choose to be empanelled voluntarily, which in turn exacerbates unequal access to health services and weakens accountability mechanisms [32]. Private corporate stakeholders are increasingly influencing India’s governance structures, thereby shifting public resources to profit-based endeavours rather than improving public healthcare infrastructure [32].

Further, India’s regulatory capacity over the health insurance sector is weak. The Clinical Establishments Act, 2010, was enacted to regulate public healthcare providers and has been implemented poorly and unevenly across the country [32]. The potential of PGHISs to exacerbate existing socio-economic inequalities is high, without proper regulation of healthcare service prices, service quality, and ethical standards [6].

A review of the literature on India’s health insurance system reveals a complex interplay between coverage expansion and historical structural exclusions. While PFHISs like PMJAY and RSBY have successfully increased insurance coverage and improved access to hospitalisations for many marginalised households [30,31], gender, caste, geography, age and chronic illness still form grounds for inequitable healthcare access [7,29].

Private sector prominence, lack of investment in public healthcare infrastructure, exclusions related to outpatient care, preventive care, and chronic illnesses, as well as poor regulation, limit the scope and effectiveness of India’s health insurance frameworks [6,32]. To move towards an equitable and successful UHC model, the literature suggests that increased public investment in healthcare, strengthened regulations for private healthcare providers, gender- and age-cognisant inclusion, and coverage of outpatient and chronic care are necessary [8,28]. Therefore, as evident above, most literature on health insurance in India examines gender, age, or chronic illness separately, rather than analysing how different exclusions compound healthcare access issues [6,7]. This is identified as a gap in the literature in this article and is the main subject of enquiry and analysis.

## 3. Methodology

This study adopts a qualitative comparative policy analysis and a documentary analysis approach to review and compare exclusions across healthcare insurance policies in India. Both primary and secondary sources are used to identify inclusion and exclusion patterns in governmental schemes (central and state) and in public and private insurers. These exclusions focus on three areas: age, gender, and pre-existing conditions that appear across policies and directly affect access to healthcare. The exclusions are analysed with respect to their impacts on access to healthcare services. The study adopts a comparative approach to identify similarities and differences across policies and implementation models.

### 3.1. Selecting Insurance Policies

The study uses a purposive sampling approach to select representative insurance policies from the Indian health insurance landscape. The sample includes:

- The central government scheme, Ayushman Bharat Pradhan Mantri Jan Arogya Yojana (AB-PMJAY),
- Thirteen state and union territory (UT) health insurance schemes,
- Four public sector insurance companies’ policies
- Five private insurance companies’ policies.

The state and UT schemes selected include those of Arunachal Pradesh, Assam, Bihar, Delhi, Gujarat, Himachal Pradesh, Jammu and Kashmir and Kerala, Maharashtra, Meghalaya, Mizoram, Nagaland, and Puducherry. The selected states/UTs were chosen to account for geographical and demographic diversity, being from locations across India. Further, insurance policies were chosen:

- to cover both publicly funded and private insurance schemes,
- to reflect different implementation models, such as trust-based, insurance-based and hybrid,
- as their policy documents and scheme guidelines are publicly available
- to reflect common industry practices, especially after the introduction of the IRDAI Guidelines (2019), which bring uniformity in exclusions.

### 3.2. Eligibility Criteria

The study includes:

- publicly funded central government health insurance schemes,
- state and UT health insurance schemes,
- public and private sector health insurance policies
- policy documents, scheme guidelines, and publicly available insurance wording documents
- policies that came into operation after the 2019 IRDAI regulatory guidelines,
- secondary literature published between 2000 and 2025 on the subjects of health insurance and access to healthcare in India.

The study excludes:

- loan-linked insurance products
- life insurance and social security schemes that do not pertain to healthcare coverage,
- employment-related schemes that are not comparable to health insurance policies
- documents without sufficient details to conduct data analysis
- Policies or materials are unavailable in the public domain.
- Accident insurance and critical illness insurance offerings, non-health insurance offerings

Secondary literature was reviewed using repositories including JSTOR, Google Scholar, and HeinOnline. Database and document searches were undertaken between July 2024 and May 2025. Search keywords included

- “health insurance” and “India”
- “health insurance exclusions” and “ India”
- “pre-existing diseases”
- “insurance waiting period”
- “out of pocket expenditure”
- “health insurance and gender”
- “health insurance for elderly persons”
- “vaccination coverage” and “insurance”
- “reproductive healthcare exclusions”
- “access to healthcare
- Health insurance for the elderly, “out of pocket expenses, “waiting periods in health insurance, “health insurance and gender, “health equity”

The literature review included peer-reviewed journal articles, books and chapters, public health and policy reports, government publications, and organisational reports. After removing duplicate, outdated, and irrelevant documents, the remaining relevant materials were reviewed to identify provisions relating to age-based exclusions, gender-based exclusions, reproductive healthcare and pre-existing ailments.

A staged process was used to screen primary and secondary data. The first stage involved a preliminary database search, and a grey literature search was conducted. A review of titles, sources, publication relevance, and relationships to health insurance exclusions and healthcare access in India. Duplicate documents were removed, and a total of 143 sources were identified as most relevant, particularly if they addressed health insurance, health finance, exclusionary practices in health insurance, and universal access to healthcare. Thematic synthesis was employed to organise the literature into themes, and a narrative review was used rather than a systematic review. Narrative reviews guided by PRISMA are well established in health policy-related research, especially where the research question concerns system-level structures.

This study also employed qualitative document analysis to identify exclusionary provisions in health insurance policies. Policy documents were reviewed and categorised to identify common exclusions related to age, gender, reproductive healthcare, and pre-existing conditions. The analysis compared patterns of exclusion across various schemes and focused on the intersecting effects of exclusions on marginalised insured, such as infants, children, women, pregnant persons, elderly persons, persons with the capacity for pregnancy and those with pre-existing conditions.

### 3.3. Limitations of the Study

This study is limited to publicly available policy documents. It does not examine how exclusions are implemented in practice during the processing of insurance claims or the provision of healthcare services. The policy sample in this study is diverse but not exhaustive of all health insurance schemes operating in India. Additionally, many state schemes do not provide consolidated policy documents, necessitating reliance on publicly available scheme guidelines and materials. The study is also limited to publicly available English-language literature. It focuses primarily on publications after 1991, although some older literature was used for historical context, potentially introducing variations in methodological rigour across policies. Since it is a narrative synthesis rather than a systematic review, the findings are subject to the author’s interpretative judgement and cannot be standardised.

## 4. Analysis: Exclusions Across Public and Private Health Insurance Policies in India

The study analyses thirteen State and Union Territory health insurance schemes (Table 1), four public-sector health insurance policies (Table 2), and five private-sector health insurance policies (Table 3).The exclusions identified across these policies are summarised below.

### 4.1. Age-Based Exclusions

Elderly persons do have unique health needs and, in particular, show increased rates of hospitalisation [36]. In India, only more affluent senior citizens showed an increased likelihood of hospital admission, while poorer elderly persons were constrained to rely on their families for healthcare costs [37]. Therefore, especially in the context of changing demographics, demand for healthcare is increasing [37]; health and well-being are critical for senior citizens; and universal health coverage should include social care, acute and chronic healthcare, and emerging needs in the later years of life [12]. However, age-based differentiation in health insurance schemes is evident in both public and private policies, affecting access to many routinely needed services for older persons, as summarised in the Table 4 below.

#### 4.1.1. AB-PMJAY

AB-PMJAY excludes outpatient care and conditions that do not require hospitalisation. While this does not seem to be an ageist exclusion on the surface, research suggests that the demand for outpatient care is substantially higher than for inpatient care among older adults [38].

It also excludes any dental treatment or surgery that is corrective, prosthetic or cosmetic, unless in certain specified situations [39]. The exclusion of corrective dental care might affect older people more than other demographics. A 2022 study by Ghosal et al. reports patterns of frequently occurring oral morbidities among those aged 45 years and above and recommends including dental healthcare in Ayushman Bharat, along with other healthcare infrastructure in the country, to meet the dental health needs of the elderly population [40].

AB-PMJAY further excludes vaccinations and immunisation, which particularly impacts infants and young children. Children who do not receive the necessary vaccinations are at elevated risk of death, morbidity, and socioeconomic vulnerabilities that limit their development over the course of life [41].

#### 4.1.2. State Health Insurance Policies

Age-based inclusions have been introduced by states such as Himachal Pradesh (Mukhya Mantri Himachal Health Care Scheme), which exempt senior citizens (aged 70 or above) from paying any premium. This scheme also imposes a differential premium for certain categories of women, including “unmarried women” aged 40 or more (INR 365/year) [42]. Similar inclusions can also be observed in states such as Kerala, where senior citizens (aged 60 years and above) can benefit from the Senior Citizen Health Insurance Scheme, which provides an additional INR 30,000 per beneficiary [43].

Arunachal Pradesh’s health insurance policy, Chief Minister Arogya Arunachal Yojana, also excludes dental treatment unless hospitalisation is required for treatment and vaccination [44]. Arunachal Pradesh has not covered conditions that do not require hospitalisation; these primarily include outpatient procedures such as cataract and dialysis. Cataract is the leading cause of bilateral blindness in India. 50 to 80% of the bilaterally blind persons in the country suffered from cataract [45]. A recent study showed that cataract prevalence amongst older persons in India stood at 14.25% as of 2024, with disparities across socioeconomic indicators [46]. However, despite these alarming statistics, most cataract patients are unable to access treatment due to economic constraints. The procedure’s high costs and the patients’ limited income result in fewer people undergoing treatment.

A study in Chennai reported that 49 respondents would refuse free surgery for cataracts owing to considerations of financial constraints and lck of additional support [47]. Similarly, excluding dialysis disadvantages a significant portion of the population. A 2018 report found that 87% of dialysis patients had to spend over 100% of their income on treatment in public facilities and 79% in private facilities [48]. Further, outpatient procedures increased households’ Out-of-Pocket Expenditures (OOPEs), causing financial distress. OOPE pushes 7% of India’s population into poverty annually, adversely affecting the older population [49].

Dental procedures are a fundamental component of geriatric care. Poor oral health can affect not only oral and general comfort but also cognition, behaviour, quality of life, and life expectancy [50]. A 2022 study found that although 77% of participants aged 45 or older had oral health issues, only 3% had dental insurance [40].

Excluding vaccinations and inoculations significantly impacts infants and children. Those who are not adequately vaccinated face higher risks of death, illness, and socioeconomic vulnerabilities, which can hinder their development [41]. Additionally, failure to cover vaccination is likely to reduce India’s low inoculation rates further. Currently, only 60% of all children are fully vaccinated [51]. There is a disparity in vaccination rates between urban and rural areas. Slow adoption of immunisation programs and limited access have led to low vaccination coverage in rural regions [51]. Excluding vaccination from the state insurance scheme will worsen this situation by increasing financial strain on households and lowering children’s immunity.

Kerala is notable for its health insurance coverage, which covers people across age groups and has no exclusions based on age or gender. The state health insurance scheme, Karunya Arogya Suraksha Padhathi, also covers all pre-existing ailments [43].

#### 4.1.3. Other Public and Private Health Insurance Policies

The IRDAI 2019 Guidelines [52] impose waiting periods for the coverage of pre-existing diseases (Excl01) and for certain other specified diseases (Excl02), depending on the insurance company concerned. The Guidelines exclude “custodial care either at home or in a nursing facility for personal care such as help with activities of daily living such as bathing, dressing, moving around either by skilled nurses or assistant or non-skilled persons” (Excl05), which is mirrored across the health insurance policies offered by both state owned and private insurance companies. The 2019 Guidelines also exclude “existing diseases” such as and loss of hearing.

The individual and group health insurance policies offered by companies such as IFFCO-Tokio [53] impose a 12-month waiting period for “cataract” and “non-infective arthritis”, among other age-related diseases such as osteoporosis. The prevalence of osteoporosis in India is a direct consequence of an up to 80% Vitamin D deficiency amongst the population, and hip fracture incidence amongst Indians occurs almost one decade before corresponding Western populations [54]. In 2015, about 230 million Indians over 50 years of age had osteoporosis [54].

Other age-based exclusions in the policies that particularly affect the geriatric population include refractive errors; custodial care (either at home or in a nursing facility for personal care); and the provision of hearing aids, spectacles, or contact lenses. Since about 20% of geriatric persons have mental health issues, including neurological disorders, these exclusions can significantly impact their quality of care [55]. Companies such as National Insurance Company Limited also exclude growth hormone therapy, which is essential for the treatment of children with growth deficiency and can be particularly harmful to their growth and development. In a similar vein, it also excludes vaccinations, including inoculations and immunisations, for children [56].

In private health insurance policies, there are common age-based exclusions across insurance providers [57]. These include rest cure, rehabilitation and respite care, as well as costs around various aids, including spectacles, contact lenses, hearing aids, crutches, dentures, artificial teeth, and other such expenses, as well as cataract treatment [53,57,58,59]. Vaccinations and inoculations are excluded, with a limited exception in cases where they are part of a company’s policy, form part of post-bite treatment, or are medically necessary and recommended by the treating medical practitioner [58,60,61,62,63]. Age-related illnesses like Alzheimer’s disease [57] are excluded, and dental treatments that consist of cosmetic surgery, dentures, dental prosthesis, dental implants, orthodontics, or surgery of any kind are excluded, unless they occur because of accidental bodily injury to natural teeth and require hospitalisation [53,57,58,62].

The age-based exclusions specified here signify how standard health insurance policies neglect the health needs of the geriatric population. While insurance companies offer specific health insurance policies for “seniors/senior citizens,” they do not address the needs of those who cannot afford or access them. Such exclusions also underscore the range of health concerns among older people that need to be accounted for to achieve universal health coverage in India. Significantly, in a recent ruling by the Vadodara District Consumer Disputes Redressal Commission, Oriental Insurance Company Limited was ordered to cover the full medical expenses of a 61-year-old complainant who had cataract surgery. The complainant sought help from the consumer forum after the insurance company initially reimbursed only part of the surgery costs. The forum rejected the company’s argument that paying the entire amount was not “customary” and “reasonable,” asserting that insurance companies cannot determine the type and amount of medical expenses required for a specific treatment [64].

### 4.2. Gender-Based Exclusions

It is seen that women in India experience substantial inequalities, with India’s Gender Development Index being only 0.849 in 2021 [65]. Inequalities between men and women reflect in the inadequacies of public sector infrastructure, leading to poor health outcomes for women [35]. This is seen in the fact that even though overall maternal deaths have reduced, poor maternal health is still a huge issue, especially within India’s oppressed caste groups. The inaccessibility or absence of quality public health services leads women to avoid seeking healthcare because they cannot afford the out-of-pocket costs of private care [66].

The field of health economics in India has also examined gender disparities in access to and use of health insurance, with studies showing significant gender differences within government health insurance programmes in states such as Rajasthan, where women account for only 33% of hospital visits among children and 43% among elderly persons [67]. Further, between 2008 and 2012, in a government health insurance program in Andhra Pradesh, the female share of hospitalisations for “sex-neutral” conditions was only 42%, and the female share of program spending was also only 39% [66]. The relative lack of control over household budgeting and personal finances amongst women in India leads to a situation in which women are deterred from accessing healthcare services (due to costs in private healthcare facilities and poor public healthcare), as well as from accessing and utilising health insurance policies [68]. Women shoulder a higher burden of OOPs for health care services than men who have similar levels of insurance coverage, primarily because of non-coverage or limits on coverage for sexual and reproductive health (SRH) services [69].

An analysis of exclusions across health insurance policies (Table 5) being offered at different levels in the country demonstrates that the highest exclusions in these policies are gender-based.

#### 4.2.1. AB-PMJAY

AB-PMJAY excludes “any assisted reproductive techniques, or infertility-related procedures, unless featuring in the National Health Benefit Package list” [39].

#### 4.2.2. State Health Insurance Policies

Himachal Pradesh has a differential premium payable for “*ekal naaris*” or certain categories of women, which include “widows, divorced, legally separated and unmarried women more than 40 years of age” [42]. The fact that the state seeks to support single women in such a way not only differentiates single women from other persons but also encourages the view that such women are more vulnerable than married women or other single persons generally and that they need support, since they are single.

States such as Gujarat [70] and Jammu and Kashmir [71], akin to the Centre, also exclude assisted reproductive techniques and infertility-related procedures. In India, about 10–15% of married couples are said to experience infertility [72], which can result in a discriminatory effect against persons with the capacity for pregnancy who are unable to conceive and wish to seek different avenues for having children. “Fertility-related procedures” are also excluded from the state insurance policy of Arunachal Pradesh [44].

Gender-based exclusions in Meghalaya [73] and Jammu and Kashmir’s [71] insurance policies, akin to PMJAY, include “assisted reproductive techniques, or infertility-related procedures”. These are also reflected in Gujarat’s policy [70]. The health insurance schemes from Maharashtra [74] and Andhra Pradesh [75] are notable here as they do not make any gender-based exclusions, and the latter includes “obstetrics and gynaecology”. The state scheme for implementing Ayushman Bharat in Bihar, launched by the Bihar Swasthya Suraksha Samiti, excludes outpatient care and assisted reproduction but includes pre-existing conditions and “newborn care” [76].

#### 4.2.3. Other Public and Private Health Insurance Policies

The 2019 Guidelines also exclude reproductive health services, in that expenses related to birth control, sterility and infertility are excluded (Excl17). These include any contraception, sterilisation, assisted reproductive services including artificial insemination and advanced reproductive technologies such as IVF, ZIFT, GIFT and ICSI, gestational surrogacy and reversal of sterilisation [52]. Such exclusions have harmful implications for women and gender diverse persons in that they result in inequity in access to healthcare for them. They can also seem to privilege traditional forms of conception over others, which can especially affect those who are unable to conceive by traditional means and wish to explore other avenues such as surrogacy or IVF. Excl18 in the Guidelines also excludes medical expenses traceable to childbirth (including complicated deliveries and caesarean sections incurred during hospitalisation) except ectopic pregnancy, as well as expenses related to miscarriages (unless due to an accident) and abortion services [52]. Exclusions relating to pregnancy and the provision of abortion services disproportionately affect women and persons with the capacity for pregnancy.

Existing diseases allowed to be permanently excluded under the Guidelines include HIV/AIDS, among others. This is alarming, since as of 2024, there are still 2,561,000 people living with HIV, with 44% being women [77]. The insurance model states that Mizoram and Nagaland had an adult HIV prevalence of more than 1%, and Manipur had an 0.81% prevalence of adult PLHIV [77]. The policies analysed by Star Health and Allied Insurance, ICICI Lombard, and IFFCO Tokio exclude “venereal and sexually transmitted diseases”, except HIV/AIDS in some cases (such as Star Health and Allied Insurance) [58,59,63].

The exclusion of STIs and AIDS from health insurance coverage exacerbates the social stigmatisation of such illnesses, severely impacting the persons suffering from them and hampering their full participation in public life. HIV-related stigma acts as a practical barrier to preventative and progressive medical care, with PLHIV often compromising on accessing care due to fear of being discriminated against [78,79]. Stigma among healthcare providers may also delay or even deny treatment, reinforcing existing barriers to equitable access to health care [79].

Exclusions are also in the stigmatisation of professions such as sex work. Sex workers already face stigma due to several factors, such as criminalisation of sex work, taboo around sex itself, narratives of rescue and rehabilitation concerning sex workers and the perceived impression of sex workers as PLHIV. Studies show that sex workers are at the receiving end of discriminatory treatment in healthcare institutions and avoid disclosures or even seeking treatment for fear of such situations [79,80]. The exclusion of reproductive health services, such as those related to pregnancy, abortion services, miscarriages and fertility, among others, also particularly affects persons with the capacity for pregnancy and is discriminatory against them. The promise of universal health coverage in India, aspired to by Ayushman Bharat, cannot be realised without including such services.

Thus, gender-based exclusions are prevalent in both state and private health insurance policies. The routinely rampant exclusion of reproductive and other health services concerning women propagates the view that such services are unimportant and can be excluded from government and private health schemes and is discriminatory to women and persons with the capacity for pregnancy.

### 4.3. Pre-Existing Ailment Exclusions

Pre-existing ailments are medical conditions diagnosed before an individual purchases or avails of an insurance policy. Pre-existing diseases often require mandatory disclosure at the time of availing an insurance policy. They may also entail a mandatory waiting period (which could stretch into years), during which the insurance policy does not cover insurance expenses. Further, health insurance premiums can increase if pre-existing conditions are disclosed, as insurers are aware that the insured person is more likely to make a claim in such circumstances. The exclusions identified across these policies are identified in Table 6 below.

#### 4.3.1. AB-PMJAY

AB-PMJAY covers all pre-existing ailments and does not impose waiting periods for the same. Beneficiaries can avail themselves of free hospitalisation and medical treatment for such illnesses. However, specific exclusions have been identified in state and private health insurance policies.

#### 4.3.2. State Health Insurance Policies

Congenital anomalies are most routinely excluded from health insurance coverage across state schemes. Some commonly prevalent congenital anomalies include Congenital Heart Disease (CHD) and epilepsy, which are excluded from health insurance policies. In India, CHD affects about 8–12 children per 1000 live births, which in turn affects mortality and morbidity [81]. Epilepsy also affects between 3 and 11.9 persons per 1000 people, with greater prevalence in rural areas and socio-economically marginalised groups [72].

While congenital internal anomalies are largely covered, congenital external diseases, defects, or anomalies are excluded from the policies of Puducherry [82], Mizoram (Bajaj Alliance) [83], and Jammu and Kashmir and Ladakh [71]. Some of these policies provide an exception to maintain functionality in cases of such diseases (as in Mizoram) [83].

#### 4.3.3. Other Public and Private Health Insurance Policies

The IRDAI Guidelines of 2019 allow private companies to impose specific waiting periods for expenses related to the treatment of pre-existing diseases and their direct complications (Excl01), and insurers may choose to exclude such expenses until the expiry of a specified number of months of continuous coverage by the insured. Pre-existing diseases are generally excluded in such policies using waiting periods. However, exceptions are laid down for when persons with pre-existing diseases may be covered (if the insured has been covered under their own or under another health insurance plan in India continuously, without lapses). Other permanently excluded diseases in the IRDAI Guidelines include epilepsy and congenital heart disease [52].

The National Insurance Company Limited, United India Insurance Company Limited, and the Oriental Insurance Company Limited exclude congenital external diseases from their individual and family health insurance policies. However, these diseases are covered after 48 months under individual and group policies issued by the New India Assurance Company Limited. Similarly, private companies such as Bajaj Allianz, Star Health and Allied Insurance, HDFC Ergo, ICICI Lombard, and IFFCO-Tokio also exclude congenital external diseases in their individual and group health insurance policies.

IRDAI Guidelines of 2019 outline specific provisions for managing pre-existing conditions within both public and private health insurance policies in India. While waiting periods are generally enforced to exclude such conditions temporarily, exceptions apply to those with continuous prior coverage. Additionally, certain conditions, such as epilepsy and congenital heart disease, are permanently excluded. Public insurers typically exclude congenital external diseases, though some offer coverage after a specified period and private insurers follow comparable practices.

An overview of the exclusions in Government, public, and private insurance programmes is provided below in Table 7 and Table 8.

## 5. Discussion

### 5.1. Comparison of Policies from Least to Most Restrictive

A comparison across the Ayushman Bharat Pradhan Mantri Jan Arogya Yojana, state health insurance schemes, and insurer-led policies reveals a clear hierarchy in the strictness of exclusions. The central scheme and certain trust-based state schemes, particularly those in Andhra Pradesh and Kerala, are more inclusive, providing coverage for pre-existing conditions without waiting periods, whereas insurer-led policies often impose waiting periods. They also have no or limited age-based exclusions. In contrast, insurance-model states such as Meghalaya, Nagaland, and Mizoram uniformly have age exclusions, demonstrating a more restrictive approach. 

Hybrid schemes in Gujarat and Maharashtra occupy an intermediate position, with age-based exclusions and pre-existing condition restrictions that partially align with insurance company-led practices. Insurance-model state schemes and all public and private insurer policies are the most restrictive, with their waiting periods for pre-existing conditions, age-based, reproductive and gender-related exclusions. For instance, all listed private insurers (Bajaj Allianz, Star Health, HDFC ERGO, ICICI Lombard, IFFCO Tokio) have the same exclusions, which are highly standardised and therefore restrictive.

### 5.2. Intersecting Exclusions and Access to Healthcare

Exclusions in insurance policies do not operate in a vacuum. Their intersecting effects can result in uneven access to healthcare across different populations. For infants and geriatric individuals, outpatient care and vaccinations are excluded in the central scheme and in multiple state schemes (e.g., Arunachal Pradesh, Bihar). Insurance-model states and insurers uniformly report age-based exclusions, indicating that preventive and primary care services are widely excluded from coverage. Specific to older persons, insurers commonly exclude or limit coverage for age-related conditions such as cataracts and impose waiting periods. 

Pregnant persons are similarly affected. Reproductive services are excluded across nearly all policy types, including the central scheme, multiple state schemes, and all insurer-led policies. This indicates systematic exclusion rather than a selective limitation of pregnancy-related and reproductive care. In multiple states, individuals requiring gender-affirming care face explicit exclusions, including Mizoram and Puducherry. Further, private insurers similarly include gender-related exclusions, again, indicating a systematic removal of these essential services from the scope of insurance schemes.

Coverage distribution is highly disparate for individuals with pre-existing conditions. While the central scheme, Andhra Pradesh, and Kerala cover pre-existing conditions, most other state schemes and all insurer-led policies indicate delayed or restricted access.

Finally, even though the central scheme targets economically marginalised populations, policy exclusions can significantly affect access to healthcare. Some vulnerabilities may result from exclusions relating to outpatient care and preventive services. This shows that coverage does not fully eliminate the need for out-of-pocket expenditure, even within targeted schemes. 

The implications of insurance exclusions on public health are significant. Firstly, excluding services such as outpatient care and vaccinations can affect preventive care across populations, as initiatives like immunisation programmes are affordable public health interventions [84]. Excluding outpatient care coverage can also affect early detection and disease treatment, thereby preventing more chronic or severe health issues [85]. Insurance exclusions for such essential and widespread public health services can, in particular, undermine access for marginalised populations.

### 5.3. Analysis of Exclusions in Health Insurance Infrastructure in India

Studies have shown that relying on private healthcare (whose costs are steadily increasing) and having inadequate medical insurance are important factors that can push people into poverty [86] and discourage the use of healthcare services [86]. Therefore, the importance of medical insurance in offsetting healthcare expenditure burdens and facilitating access to healthcare is critical [87]. One key feature of health insurance plan design is to provide sufficient coverage for participants’ unexpected health care needs without encouraging unnecessary spending.

It is seen that when insured individuals pay out of pocket for a smaller share of their medical care costs, they are likely to consume more care—a phenomenon known as “moral hazard” [88]. Further, when individuals select a specific plan from among various options, those who are more likely to require care generally choose plans with greater coverage—a phenomenon known as “adverse selection” [87]. Therefore, someone who spends more in generous plans could do so because of the plan’s enhanced coverage, or because such a plan attracts individuals who have more underlying health requirements.

The term “moral hazard” originated in the 19th and early 20th centuries when people did not trust insurers [88]. The general impression of insurers was that they were “gambling on the bad luck or ill fate of others”—and in response to this perception, insurance agents and promoters turned back the focus on distrustful persons, “screening out those who posed a ‘moral hazard’ to honest policyholders seeking to cover genuine risks” [89]. Insurance agents, who were primarily male, white and middle-class, were trained to screen out such ‘distrustful risks’ (mainly from ethnic minorities, immigrant groups and other such communities) and “take on the mantle of moral integrity” [89].

However, the meaning of ‘moral hazard’ changed over time, and now the implication is that “if policyholders’ costs drop to zero with single payer, publicly funded universal health insurance, demand and expenditures would become infinite” [89]. Therefore, this theory casts doubt on the merit of any health insurance, and the ‘moral hazard’ is invoked in response to any campaign or initiative seeking universal health insurance.

The ‘moral hazard’ argument used by insurance companies can be seen to form part of the rationale for exclusions in insurance policies. Further, the moral hazard faced by policyholders seeking coverage for medical expenses arises from insurers undertaking measures such as “setting coverage limits, deductibles, and co-payments; denying legitimate claims; profiting from delayed claims processing”, which makes it challenging to get valid claims paid [89]. Further, contingent or conditional coverage provided by insurers, which can disqualify claimants from perceived eligible services, as well as easily missed fine print, such as mandatory prior notification for elective procedures, can also restrict insurance coverage in ways that adversely impact complainants.

### 5.4. Health Insurance Policy Exclusions, the Right to Health and UHC

There have been limited instances in which health insurance policy disparities have been legally challenged in the context of realising the Constitutionally protected right to health. The right to health was not originally conceived as an enforceable fundamental right. It was through judicial interpretation of Article 21 (right to life) of the Constitution of India, through a plethora of judgements, that the right to health came to be recognised as an integral part of the constitutional framework and dignified life [90]. In *Parmanand Kataria v*. *Union of India* [91], the Supreme Court affirmed the right to emergency medical aid to all individuals in India. Subsequent decisions, such as *C.E.S.C. Ltd. v*. *Subhash Chandra Bose* [92] and *Consumer Education & Research Centre v*. *Union of India* [93], further expanded this understanding by connecting workers’ health to broader questions of dignity, productivity, and socio-economic justice. Notably, the Court recognised that health extends beyond the absence of illness, including compulsory health insurance coverage for workers working in hazardous industries.

Most significantly, in *LIC of India v*. *Consumer Education & Research Centre* [94], the Supreme Court held that, in the context of contractual state action, the State is obligated to act justly, fairly, and reasonably in the public interest, aligned with the constitutional conscience and socio-economic justice. The Court stated that LIC’s insurance policies and their terms involve a public element and held that every action of a public authority should be guided by public interest.

One such challenge was raised before the Delhi High Court in 2018, in *M/S. United India Insurance Company Limited v. Jai Prakash Tayal* [95], where the petitioner had been refused a claim availed from a Mediclaim policy, based on the exclusion of ‘genetic disorders’. The petitioner had been hospitalised for Hypertrophic Cardiomyopathy (HCM) in 2004, and his claim was honoured by the insurer in 2006 [95]. Thereafter, when the petitioner made a claim in 2011, it was rejected under a genetic exclusion clause [95].

Despite the respondent being a private insurer, the Delhi High Court examined the petition in the background of Articles 14 (right to equality) and 21 (right to life and liberty) of the Constitution, with the main question being whether lawful discrimination was allowed for persons with a genetic disorder under health insurance [95]. The Court held that medical care is a basic human right, with health insurance being an integral component. The Court held that discrimination against individuals based on ‘genetic disorders’ would be violative of their fundamental right to health [95].

The Court notably stated that insurance companies must structure their contracts to satisfy the factors of ‘reasonableness’ and ‘intelligibility’ (based on empirical testing and information), without being arbitrary or exclusionary, and without relying on vague or interpretative factors [95]. Thereafter, the Madras High Court [96] passed a similar judgment, upholding the petitioner’s claim challenging the genetic exclusion clause in their insurance policy.

This judicial engagement with genetic exclusions in health insurance policies (even those of private actors) indicates that such exclusions must be implemented in a manner that is non-exclusionary and non-discriminatory. This is in line with the Principles of UHC, the first of which is ‘universality’ [97]. ‘Universality’ prescribes that the UHC system must be “genuinely universal in its scope”, with persons of all socio-economic classes having access to quality and affordable healthcare. Further, the principle of ‘non-exclusion and non-discrimination’ clearly states that, as per the ‘universality’ principle, “no person should be excluded from services or benefits on grounds of current or pre-existing illnesses and health conditions” or because there is a special health service requirement [97]. The UHC principles also prohibit discrimination or exclusion based on identity characteristics, including ‘occupation’ or ‘other social or personal background’. The limitations of the study are highlighted in the methodology section.

### 5.5. Observations

In sum, the research identifies patterns that reveal healthcare access in India is not uniformly structured and is fragmented in terms of inclusion and exclusion. One key finding is that gender-based exclusions are consistent across policies. Nearly all policies, whether central, state or insurer-led, include reproductive exclusions. This suggests that such exclusions are embedded across the system. At the same time, trust-based schemes have reduced exclusions, particularly for pre-existing conditions and age-based services, indicating that broader coverage is feasible.

Further, policies are standardised in accordance with IRDAI regulations. Insurer-led policies display almost identical exclusions, especially for pre-existing conditions and reproductive services. These findings show that even if overall insurance coverage is expanded, equitable access to healthcare cannot be achieved without undertaking an intersectional approach towards revising the structure and scope of policy exclusions. Exclusions should not be a matter of policy design but rather central mechanisms that shape access across different populations and jurisdictions.

The research findings have significant implications for health insurance coverage policies in India. Firstly, the overall scope of exclusions must be reconsidered in light of UHC principles of universality, non-exclusion and non-discrimination, as well as established jurisprudence on the right to health. From a public health perspective, removing exclusions for outpatient services, vaccinations, and immunisations can reduce disease burdens across the country and improve statistics on infant/child morbidity and mortality.

Secondly, jurisprudence establishes the unconstitutionality of genetic exclusions, which must be reviewed and eliminated, especially when they interfere with universal access to healthcare treatment. Pre-existing conditions, like congenital anomalies, should not be grounds for blanket exclusion from healthcare policies, and the Delhi High Court’s order in *Jay Parkash Tayal* [95] clearly situates even private insurers’ health policies within constitutional rights frameworks. Policies around gender-based exclusions like reproductive care must also be reviewed, as these standardised exclusions can have negative implications for access to healthcare by women, children and elderly citizens.

Age-based exclusions in health insurance policies often apply to conditions that are age-related or more prevalent in certain age groups, such as arthritis and cataracts. Additionally, exclusions may extend to the provision of aids such as cochlear implants and crutches, thereby impacting the quality of healthcare services for individuals in need. Many policies also exclude vaccinations, immunisations, and growth hormone therapy, significantly affecting infants and young children and potentially hindering their growth and long-term health.

Gender-based exclusions, as observed in the policies analysed, are generally consistent across policies and more numerous compared to exclusions based on age and pre-existing ailments. These exclusions encompass maternity care (referred to as “childbirth” in some policies), pregnancy-related services (including miscarriages and abortion), sterility, fertility/infertility treatments, assisted reproductive services (e.g., surrogacy, IVF), and contraception (or birth control). Additionally, gender affirming procedures (e.g., “change of sex” treatments, hormone replacement therapy for sex change) are universally excluded in all policies. Other exclusions pertain to sexually transmitted infections, referred to as “sexually transmitted diseases” or “venereal diseases” in policy documents.

Many health insurance policies exclude congenital external anomalies as “pre-existing” ailments. In contrast, others impose specific waiting periods (often after 48 months from the start of the insurance policy) before covering such ailments. The categories of age, gender, and pre-existing ailments are not strictly delineated and frequently intersect across policies. For example, the differential premium payable by “unmarried women over 40 years of age” under the state health insurance policy of Himachal Pradesh may be perceived as an inclusion under the age criterion (as it provides benefits for citizens over 40 years) but as an exclusion under gender (as it implies that single women need government support if not provided by their spouses).

The wide exclusions across age, gender and pre-existing conditions in public and private insurance policies show that the fulfilment of Universal Health Coverage in India is severely hampered at the policy level. The AB—PMJAY’s age-related restrictions, the ubiquitous restrictions on SRH services across policies, as well as the waiting periods prescribed by private insurance companies for pre-existing ailments, widely restrict access to healthcare for various vulnerable groups. The exclusions across policies reflect the state’s inability to address social and economic inequities by furthering access to essential healthcare for all. On the contrary, these exclusions will almost certainly entail greater OOP expenditure for persons seeking treatment, further restricting access to healthcare for poorer and marginalised groups. As Qadeer et al. caution that the contemporary UHC are largely driven by insurance-based and market-oriented approaches. Yet, it is imperative to strengthen the public health system and focus on access to comprehensive care, not merely universal insurance coverage [98].

## 6. Concluding Observations

Wide exclusions across age, gender and pre-existing conditions in public and private insurance policies show that fulfilment of UHC in India is severely hampered at the policy level. Age-related restrictions, ubiquitous restrictions on SRH services across policies, and waiting periods prescribed by private insurance companies for pre-existing ailments widely restrict access to healthcare for vulnerable groups. The exclusions reflect the state’s inability to address social and economic inequities by furthering access to essential healthcare for all. These exclusions almost certainly entail greater OOPE for persons seeking treatment, further restricting access to healthcare for poorer and marginalised groups. Therefore, until these exclusions are addressed, UHC’s goals remain illusionary.

Challenges of achieving UHC are compounded by broader failures in access to health care for citizens, driven by intersecting socioeconomic factors and geographical disparities across the country. Disparities in health care access and insurance utilisation are heavily predicated on economic status, with intersecting factors like gender, caste, indigeneity, and disability also playing a significant role in the accessibility of healthcare services. Public health infrastructure across India remains woefully inadequate and unequally distributed, further impeding access to healthcare services. Until these broader barriers are addressed, achieving health equity may not be a realistic goal.

## Figures and Tables

**Table 1:** State and Union Territory Health Insurance Policies.

Chief Minister Arogya Arunachal Yojana	Arunachal Pradesh	2018	Active	Scheme Guidelines (Latest Available)	Trust
Ayushman Asom Mukhya Mantri Scheme	Assam	2016/2018	Active	Consolidated scheme guidelines	Trust
Bihar Swasthya Suraksha Samiti Scheme	Bihar	2018	Active	Scheme documents aligned with AB-PMJAY	Trust
Delhi Arogya Kosh	Delhi	2017	Active	Latest scheme guidelines	Trust
Mukhyamantri Amrutam Scheme	Gujarat	2012	Active (expanded)	Post-expansion guidelines	Hybrid
Himachal Health Care Scheme	Himachal Pradesh	2016	Active	Latest scheme guidelines	Trust
Jammu and Kashmir Health Scheme	J&K	2019	Active	Insurance contract (post-2019)	Insurance
Karunya Arogya Suraksha Padhathi	Kerala	2019	Active	Latest scheme guidelines	Trust
Mahatma Jyotirao Phule Scheme	Maharashtra	2012	Active (modified)	Revised scheme guidelines	Hybrid
Megha Health Insurance Scheme	Meghalaya	2013	Active	Insurance-based policy documents	Insurance
Mizoram State Scheme	Mizoram	2019	Active	Post-AB-PMJAY integration	Insurance
Nagaland Health Scheme	Nagaland	2018	Active	Latest insurance policy	Insurance
Puducherry Scheme	Puducherry	2018	Active	AB-PMJAY aligned version	Insurance

“Status” refers to the policy’s current operational status.

**Table 2:** Public Sector Health Insurance Policies.

S. No.	Company	Type	Status	Policy Version Analysed	Implementation Model
1	National Insurance Company Limited	Public insurer	Active	Individual health insurance policy (post-2019 version)	Insurance
2	United India Insurance Company Limited	Public insurer	Active	Latest publicly available health policy (post-2019 format)	Insurance
3	The New India Assurance Company Limited	Public insurer	Active	New Mediclaim policy wording post-2019	Insurance
4	The Oriental Insurance Company Limited	Public insurer	Active	Standard health insurance policy document compliant with post-2019 IRDAI guidelines	Insurance

‘Status’ refers to the current operational status of the insurance company.

**Table 3:** Private Sector Health Insurance Policies.

S. No.	Company	Type	Status	Policy Version Analysed	Implementation Model
1	Bajaj Allianz General Insurance Company Limited	Private insurer	Active	Comprehensive health insurance policy (post-2019 IRDAI-compliant)	Insurance
2	Star Health and Allied Insurance Company Limited	Private insurer	Active	Standard individual/family floater policy wording (latest publicly available version)	Insurance
3	HDFC ERGO General Insurance Company Limited	Private insurer	Active	Latest publicly available health insurance policy (post-2019 format)	Insurance
4	ICICI Lombard General Insurance Company Limited	Private insurer	Active	Representative comprehensive health insurance policy post-2019 compliant	Insurance
5	IFFCO-Tokio General Insurance Company Limited	Private insurer	Active	Latest available health insurance policy	Insurance

‘Status’ refers to the current operational status of the insurance company.

**Table 4:** Age-Based Exclusions Across Health Insurance Policies in India.

Policy/Scheme	Outpatient Care Excluded	Vaccinations Excluded	Age-Related Diseases (e.g., Cataract)	Assistive Devices Excluded	Waiting Periods for Age-Related Conditions
Ayushman Bharat Pradhan Mantri Jan Arogya Yojana	Yes	Yes	Partial	Yes	No
State Schemes (General Pattern)	Yes (most states)	Yes (many states)	Yes	Yes	Limited
Andhra Pradesh (Dr. YSR Aarogyasri)	Limited	Limited	Limited	Limited	No
Kerala (Karunya Arogya Suraksha Padhathi)	No	No	No	Limited	No
Insurance Model States (e.g., Meghalaya, Nagaland)	Yes	Yes	Yes	Yes	Yes
Public Insurers	Yes	Yes	Yes	Yes	Yes (Excl02)
Private Insurers	Yes	Yes	Yes	Yes	Yes (Excl02)

**Table 5:** Gender-Based Exclusions Across Health Insurance Policies in India.

Policy/Scheme	Reproductive Services Excluded (Excl17)	Pregnancy/Childbirth Excluded (Excl18)	Gender-Affirming Care Excluded (Excl07)	Sexually Transmitted Infections Excluded	Differential Treatment of Women
Ayushman Bharat Pradhan Mantri Jan Arogya Yojana	Yes	Partial	Yes	Partial	No
State Schemes (General Pattern)	Yes	Yes	Yes	Yes	Limited
Himachal Pradesh Scheme	Yes	Yes	Yes	Yes	Yes
Andhra Pradesh	No	Limited	Limited	Limited	No
Kerala	No	No	No	Limited	No
Insurance Model States	Yes	Yes	Yes	Yes	No
Public Insurers	Yes	Yes	Yes	Yes	No
Private Insurers	Yes	Yes	Yes	Yes	No

**Table 6:** Pre-Existing Ailment Exclusions Across Health Insurance Policies in India.

Policy/Scheme	Pre-Existing Diseases Covered	Waiting Period (Excl01)	Congenital Conditions Excluded	Permanent Exclusions (e.g., Epilepsy)	Continuous Coverage Exception
Ayushman Bharat Pradhan Mantri Jan Arogya Yojana	Yes	No	Limited	No	Not required
State Schemes (General Pattern)	Partial	Limited	Yes	Limited	Rare
Andhra Pradesh	Yes	No	Limited	No	Not required
Kerala	Yes	No	No	No	Not required
Insurance Model States	Partial	Yes	Yes	Yes	Limited
Public Insurers	No (initially excluded)	Yes	Yes	Yes	Yes
Private Insurers	No (initially excluded)	Yes	Yes	Yes	Yes

**Table 7:** Government Health Insurance Schemes and Exclusions (Condensed).

Policy	Jurisdiction	Year	Model	Age Exclusions	Gender Exclusions	Pre-existing Exclusions	IRDAI Clauses	Post-2019
Ayushman Bharat Pradhan Mantri Jan Arogya Yojana	Central	2018	Mixed	OPD, vaccines excluded	Assisted reproduction excluded	Covered	Excl17, Excl18	No
Dr. YSR Aarogyasri Scheme	Andhra Pradesh	2007	Trust	Limited	None	Covered	Minimal	No
Chief Minister Arogya Arunachal Yojana	Arunachal Pradesh	2018	Trust	OPD excluded	Fertility excluded	Congenital excluded	Excl01, Excl17	Partial
Ayushman Asom Scheme	Assam	2016/18	Trust	Limited	Assisted reproduction excluded	Yes	Excl01, Excl17	Partial
Bihar Health Scheme	Bihar	2018	Trust	OPD excluded	Assisted reproduction excluded	Partial	Excl17	Limited
Delhi Arogya Kosh	Delhi	2017	Trust	Limited	Limited	Limited	Minimal	No
Mukhyamantri Amrutam Schemes	Gujarat	2012	Hybrid	Yes	Assisted reproduction excluded	Yes	Excl01, Excl17	Yes
Himachal Health Scheme	Himachal Pradesh	2016	Trust	Mixed	Gender-differentiated premiums	Limited	Limited	No
Jammu & Kashmir Scheme	Jammu and Kashmir	2019	Insurance	Yes	Assisted reproduction excluded	Yes	Excl01, Excl17, Excl18	Yes
Karunya Arogya Suraksha Padhathi	Kerala	2019	Trust	None	None	Covered	Minimal	No
Mahatma Phule Scheme	Maharashtra	2012	Hybrid	Yes	Limited	Yes	Excl01, Excl02	Yes
Megha Health Scheme	Meghalaya	2013	Insurance	Yes	Assisted reproduction excluded	Yes	Excl01, Excl17	Yes
Mizoram Scheme	Mizoram	2019	Insurance	Yes	Gender-affirming care excluded	Yes	Excl07, Excl17	Yes
Nagaland Health Scheme	Nagaland	2018	Insurance	Yes	Yes	Yes	Excl01, Excl17	Yes
Puducherry Implementation	Puducherry	2018	Insurance	Yes	Gender-affirming care excluded	Yes	Excl07, Excl17	Yes

**Table 8:** Public and Private Insurers and Exclusions (Condensed).

Policy	Type	Model	Age Exclusions	Gender Exclusions	Pre-Existing Exclusions	IRDAI Clauses	Post-2019
National Insurance Company Limited	Public	Insurance	Cataract, aids excluded	Reproductive exclusions	Waiting periods	Excl01, Excl02, Excl05, Excl17	Yes
United India Insurance	Public	Insurance	Yes	Yes	Yes	Excl01, Excl17	Yes
New India Assurance	Public	Insurance	Limited (waiting)	Yes	Yes	Excl01, Excl02	Yes
Oriental Insurance	Public	Insurance	Yes	Yes	Yes	Excl01, Excl17	Yes
Bajaj Allianz	Private	Insurance	Yes	Reproductive + gender exclusions	Yes	Excl01, Excl07, Excl17, Excl18	Yes
Star Health	Private	Insurance	Yes	STI + gender exclusions	Yes	Excl01, Excl07, Excl17	Yes
HDFC ERGO	Private	Insurance	Yes	Yes	Yes	Excl01, Excl17	Yes
ICICI Lombard	Private	Insurance	Yes	Yes	Yes	Excl01, Excl17	Yes
IFFCO Tokio	Private	Insurance	Waiting periods	Yes	Yes	Excl01, Excl02, Excl17	Yes

## Data Availability

The original data presented in the study are openly available as referenced.

## Abbreviations

AB-PMJAY	Ayushman Bharat Pradhan Mantri Jan Arogya Yojana.
AB-NHPM/AB-NHPS	Ayushman Bharat National Health Protection Mission/Scheme
CGHS	Central Government Health Scheme.
ESIC/ESIS	Employees’ State Insurance Corporation/Scheme
HIV/AIDS	Human Immunodeficiency Virus/Acquired Immunodeficiency Syndrome
HLEG	High-Level Expert Group on Universal Health Coverage
IRDAI	Insurance Regulatory and Development Authority of India
LIC	Life Insurance Corporation of India
NABH	National Accreditation Board for Hospitals and Healthcare Providers
NFHS	National Family Health Survey
NHA	National Health Authority
NITI Aayog	National Institution for Transforming India
NRHM	National Rural Health Mission
NSSO	National Sample Survey Organisation
OOPE	Out-of-Pocket Expenditure
PLHIV	People Living with HIV
RSBY	Rashtriya Swasthya Bima Yojana
SHA	State Health Agency
STIs	Sexually Transmitted Infections
UHC	Universal Health Coverage
